# Robot-Assisted Mirror Therapy for Upper Limb and Hand Recovery After Stroke: Clinical Efficacy and Insights into Neural Mechanisms

**DOI:** 10.3390/jcm15010350

**Published:** 2026-01-02

**Authors:** Shixin Li, Jiayi Zhang, Yang Xu, Yonghong Yang

**Affiliations:** 1 Rehabilitation Medicine Center, Institute of Rehabilitation Medicine, West China Hospital, Sichuan University, Chengdu 610041, China; 2023224020158@stu.scu.edu.cn (S.L.); jiayi@stu.scu.edu.cn (J.Z.); xyprotk@g.ecc.u-tokyo.ac.jp (Y.X.); 2 Key Laboratory of Rehabilitation Medicine in Sichuan Province, West China Hospital, Sichuan University, Chengdu 610041, China

**Keywords:** stroke, upper limb, motor dysfunction, functional near-infrared spectroscopy (fNIRS), robot-assisted therapy, mirror therapy

## Abstract

**Objective:** This study investigated the efficacy and neural mechanisms of robot-assisted mirror therapy (RMT) for post-stroke upper limb rehabilitation. RMT integrates the multimodal feedback of mirror therapy with robotic precision and repetition to enhance cortical activation and neuroplasticity. **Methods:** Seventy-eight stroke patients were randomly assigned to control, mirror therapy (MT), or RMT groups. All received conventional rehabilitation; the MT group additionally underwent mirror therapy, and the RMT group received robot-assisted mirror therapy combined with functional electrical stimulation. The primary outcome was the Fugl–Meyer Assessment for Upper Extremity (FMA-UE), with secondary measures including spasticity, dexterity, daily living, and quality of life. Functional near-infrared spectroscopy (fNIRS) was applied to assess cortical activation and connectivity at baseline, post-intervention, and one-month follow-up. **Results:** All groups showed significant time effects, though between-group differences were limited. Subgroup analysis revealed that patients at Brunnstrom stages I–II in the MT group achieved greater improvements in upper limb function, dexterity, and daily living ability. fNIRS findings showed enhanced activation in the right sensory association cortex and increased prefrontal–sensory connectivity. **Conclusions:** While all interventions improved motor outcomes, MT yielded slightly superior recovery associated with neuroplastic changes. RMT demonstrated high safety, compliance, and potential benefit for patients with severe motor deficits.

## 1. Introduction

Stroke, caused by an interruption of the blood supply or rupture of cerebral vessel in the brain, resulting in brain tissue damage [1]. Upper limb dysfunction affects 50–80% of patients in the acute phase, and nearly half of these cases become chronic disabilities [2]. Recently, Rehabilitation robotics and mirror therapy have gained significant traction worldwide for managing post-stroke upper limb impairment, with robust evidence increasing confirming their efficacy. MT, introduced in 1996 [3] for phantom limb pain and applied to hemiplegic patients in 1999 [4], has become a widely used intervention for post-stroke upper limb rehabilitation [5]. It relies on visual feedback, creating an illusion of the affected limb’s movement by mirroring the unaffected side. This illusion induces bilateral motor attempts. facilitating neural retraining. MT has been demonstrated to promote a range of functional recoveries, including improvements in muscle tone [6], motor and hand function [7,8], coordination [8], and early rehabilitation outcomes, Furthermore, it also positively impacts psychosocial factors by enhancing patient confidence and motivation [9]. The mechanisms of MT involved the activation of the mirror neuron system [10,11] and modulation of sensorimotor networks [12], which in turn stimulate activity in the occipital, parietal, and primary motor, and somatosensory cortices [13]. With repeated application, MT induces neuroplastic adaptations, including premotor reorganization [14], increased corticospinal excitability [15], improved interhemispheric communication [14,16,17], and strengthened functional connectivity within motor-related regions [18,19,20]. These neural adaptations provide a biological basis for MT’s therapeutic effects and support its application in stroke rehabilitation. Robot-assisted technology (RT), which integrates mechanical, electronic, and computer engineering, enables highly precise and repeatable upper-limb rehabilitation training for stroke patients. Compared with conventional physical therapy, RT provides more accurate movement guidance and sustains stable control over training intensity. Consequently, it effectively improves upper-limb muscle strength, joint mobility, and motor coordination [21]. Evidence from randomized controlled trials indicates that robot-assisted technology can effectively enhance upper-limb motor control, muscle strength, range of motion, and hand function [22,23], particularly in proximal segments [24], with pronounced benefits for patients with severe impairments [25,26]. In addition, RT devices facilitate real-time recording and analysis of movement data, supporting the development of individualized rehabilitation programs and the monitoring of recovery progress, thereby enhancing therapeutic efficiency and improving patient outcomes [27].

Robot-assisted mirror therapy (RMT) integrates the precise mechanical assistance of robotic therapy with the visual feedback of MT. This integration aligns external motor stimulation with patients’ voluntary motor intentions, thereby effectively promoting neural plasticity. Based on this mechanistic framework, we hypothesize that RMT should yield superior therapeutic outcomes compared with MT alone for several reasons. First, robotic assistance provides continuous, quantifiable kinematic feedback that reinforces movement execution and enhances motor learning. Second, the immersive visual feedback from MT may further amplify movement-related cortical activations while concurrently supporting patients’ psychological well-being, potentially improving engagement and compliance. Finally, the integration of RT enables real-time monitoring and individualized adjustment of training parameters, which may optimize treatment precision and long-term functional gains. Although prior studies have reported mixed findings on the efficacy of RMT, with some indicating enhanced motor coordination and others noting possible overreliance on visual feedback, these inconsistencies underscore the need for further empirical investigation. Consequently, the long-term efficacy of RMT and the optimization of individualized protocols remain critical challenges [27,28,29].

The present study aims to evaluate the effects of RMT on upper-limb functional recovery in stroke patients and to explore its underlying neural mechanisms by monitoring cortical hemodynamic changes with fNIRS. These findings are anticipated to offer novel therapeutic avenues for post-stroke rehabilitation, improve patients’ quality of life, and contribute to the advancement of public health and rehabilitation strategies.

## 2. Materials and Methods

### 2.1. Study Design

**Figure 1:**
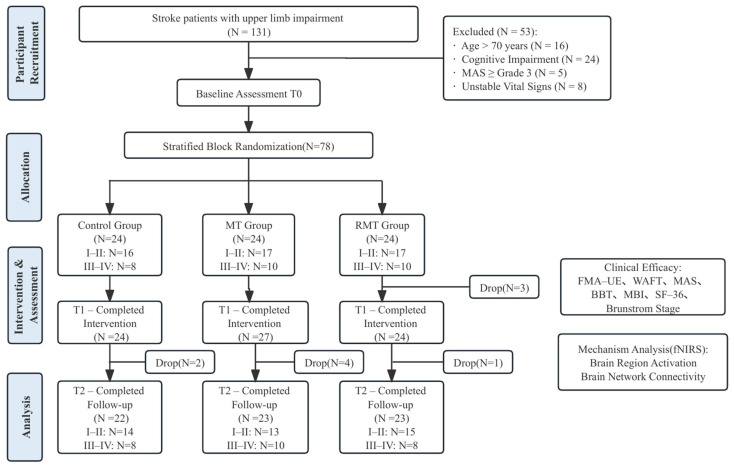
Study Flowchart.

This study was designed as a prospective, randomized, controlled, single-blind trial (assessor-blinded). Participants were randomly assigned to a control group, an MT group, or an RMT group. Assessments were conducted at baseline (T0), post-intervention (T1,3 weeks), and follow-up (T2,1 month). The trial flow diagram is shown in Figure 1.

### 2.2. Randomization and Blinding

A stratified block randomization method was applied according to Brunnstrom stages (I–II, III–IV), with six participants per block. Independent researchers generated random sequences using R 4.4.1 software and ensured allocation concealment. In case of deterioration or adverse events, eligibility was reassessed, with unblinding and ethical reporting if necessary.

### 2.3. Sample Size

The sample size was calculated using G*Power 3.1, with the FMA-UE score as the primary outcome. Assuming an effect size of 0.25, α = 0.05, and β = 0.20, the required sample size was 66. Considering a 20% dropout rate, a total of 78 participants (26 per group) were ultimately enrolled.

### 2.4. Ethics

This study was approved by the Biomedical Ethics Committee of West China Hospital, Sichuan University (No. 2024 (13)) and registered in the Chinese Clinical Trial Registry (ChiCTR2400081976).

### 2.5. Participants

The first participant was enrolled in the study on 20 March 2024. The inclusion criteria were as follows: (1) first-ever stroke confirmed by CT or MRI, meeting the diagnostic criteria in the 2019 Chinese Guidelines for Major Cerebrovascular Diseases; (2) disease duration of 2 weeks to 6 months; (3) affected upper limb at Brunnstrom stage I–IV; (4) able to maintain unsupported sitting for at least 20 min; (5) age between 18 and 70 years; (6) alert, oriented, capable of following simple instructions and stable vital signs; and (7) patient or legal guardian provided informed consent voluntarily.

The exclusion criteria included: (1) severe psychiatric disorders, hearing impairment, or visual impairment; (2) severe cognitive or perceptual impairments preventing cooperation with treatment; (3) severe cardiopulmonary, hepatic, or renal dysfunction; (4) spasticity of the affected upper limb with a Modified Ashworth Scale ≥ 3; and (5) presence of other neurological disorders affecting upper limb function.

### 2.6. Interventions

All interventions were conducted in addition to conventional rehabilitation [30], lasting 3 weeks (5 sessions per week, 20 min per session). Procedures were delivered by trained therapists in separate treatment rooms to avoid cross-group contamination.

MT Group: The protocol was based on the standardized program developed by Yavuzer et al. [7]. Participants performed progressive upper-limb tasks using a mirror box, with the unaffected limb moving in front of the mirror while the affected limb was concealed, thereby creating the illusion of symmetrical movement.

RMT Group: Participants wore a soft robotic hand rehabilitation device (RSD, Nanjing), which captured movements of the unaffected hand to drive synchronous imitation by the affected hand. Additionally, parameterized functional electrical stimulation was applied to the wrist extensor muscles of the paretic side to support motor control.

### 2.7. Clinical Efficacy and Mechanistic Indicators

#### 2.7.1. Clinical Efficacy Indicators

The primary outcome was the Fugl–Meyer Assessment of Upper Extremity (FMA-UE) [31], a widely validated scale assessing sensorimotor recovery after stroke. The FMA-UE evaluates movement synergy, coordination, joint function, and reflex activity of the paretic upper limb, with scores ranging from 0 to 66, where higher scores indicate better motor recovery.

Secondary outcomes included several standardized functional measures. The Wolf Motor Function Test (WMFT) [32] is a standardized performance-based assessment of post-stroke upper-limb motor function, consisting of 15 timed functional tasks and 2 strength tests, each rated on a 0–5 scale. The Modified Ashworth Scale (MAS) [33] evaluates muscle spasticity by rating resistance to passive stretch on a 0–4 scale, with higher scores reflecting greater spasticity. Brunnstrom stages [34] classify the degree of motor recovery based on sequential patterns of limb synergies, ranging from Stage I (flaccidity) to Stage VI (near-normal movement). The Box and Block Test (BBT) [35] measures gross manual dexterity by counting the number of blocks a patient can transfer between compartments within 60 s. The Modified Barthel Index (MBI) [36] assesses independence in activities of daily living, including feeding, grooming, dressing, transfers, and mobility, with higher scores indicating greater functional independence. The 36-Item Short Form Health Survey (SF-36) [37] evaluates health-related quality of life across eight domains, such as physical functioning, vitality, pain, and social participation.

#### 2.7.2. fNIRS Data Acquisition and Preprocessing

Cortical activation and connectivity were measured using a portable multi-channel fNIRS system (NirSmart-TB, Danyang Huichuang Medical, Danyang, China) during resting and task states. ROIs included bilateral M1, PMC, SMA, DLPFC, S1, and SAC. Data preprocessing was performed in NirSpark and comprised: (1) Marker insertion at key task points (30 s, 210 s, 390 s). (2) Conversion of raw intensity to optical density. (3) Motion artifact correction using standard deviation and spline interpolation. (4) Band-pass filtering (0.01–0.20 Hz) to remove physiological noise (respiration, cardiac, Mayer waves). (5) Conversion to HbO_2_ and HbT concentration changes using the modified Beer–Lambert law. (6) Block averaging for resting and task states. (7) Calculation of mean differences in HbO_2_ (MeandiffHbO_2_) and HbT (MeandiffHbT) between task and rest. (8) Mapping channel–ROI correspondence on a head template.

### 2.8. Statistical Analysis

Statistical analyses were performed using R software version 4.4.1, with two-tailed tests and a significance level of α = 0.05. The Shapiro–Wilk test was used to assess the normality of the data, and Levene’s test was used to evaluate homogeneity of variance. Normally distributed continuous variables were expressed as mean ± standard deviation (Mean ± SD), while non-normally distributed or categorical variables were described using median (P25, P75) or percentages. Categorical variables were compared using the chi-square test, and continuous variables (e.g., age, upper limb function scores) were compared using one-way analysis of variance (ANOVA) or non-parametric tests (e.g., Kruskal–Wallis H test) to assess baseline characteristics and ensure successful randomization. Outcome measures were analyzed using repeated measures ANOVA, with subgroup analyses performed according to Brunnstrom stages I–II and III–IV of the affected upper limbs. Per-protocol (PP) analysis was employed to include only participants who fully adhered to the treatment protocol, thereby reducing bias from protocol deviations and enhancing the internal validity of the results.

Brain activation was analyzed using two-way repeated measures analysis of variance (rmANOVA) to compare within- and between-group differences in MeandiffHbO_2_ and MeandiffHbT across the ROIs. First, the Shapiro–Wilk test was used to assess the normality of continuous data. Then, two-way rmANOVA was applied to examine within- and between-group differences of MeandiffHbO_2_ and MeandiffHbT at different time points for each ROI. Mauchly’s test of sphericity was conducted to verify the sphericity assumption of rmANOVA. When significant interaction effects were detected, post hoc comparisons were performed using Bonferroni correction to further investigate within- and between-group effects at different time points. If significant baseline differences were observed, continuous variables were included as covariates or categorical variables as factors in a repeated measures analysis of covariance (rmANCOVA).

Functional connectivity of the brain network was analyzed by calculating the Pearson correlation coefficients (r) of the HbO_2_ time series between each CH-CH and ROI-ROI during the sitting balance task, serving as an index of brain network connectivity. To normalize the distribution of correlation coefficients, the r values were transformed using Fisher’s z transformation. Subsequently, the number of connections in the network with absolute z values exceeding a predefined threshold was calculated to evaluate the relatively important connections within the network. The ratio of relatively important connections to the total number of connections in the network was then computed. Statistical analyses were conducted using rmANOVA or rmANCOVA. When significant interaction effects were observed, post hoc comparisons were performed with Bonferroni correction to examine within- and between-group effects at different time points.

## 3. Results

### 3.1. Basic Characteristics of Participants

Baseline characteristic analysis (Table 1) showed that there were no significant differences were observed among the three groups for majority of baseline variables assessed (e.g., age, sex, marital status, educational level, stroke type, lesion location, hemiplegic side, disease duration, hypertension, hyperlipidemia, diabetes, and alcohol consumption) (*p* > 0.05). However, smoking habits differed significantly among groups (*p* = 0.013), with a higher proportion of smokers in the control group (45.5%) compared to the MT and RMT groups (both 13.0%).

### 3.2. Clinical Efficacy

Results, as shown in Table 2, demonstrated significant time effects across all groups for multiple measures including FMA-UE, WMFT, MAS (elbow flexion/extension), Brunnstrom staging (upper limb/hand), BBT, MBI, and SF-36 at different timepoints. FMA-UE analysis revealed significant main effects of time (F = 192.2, *p* < 0.001) but nonsignificant group (F = 0.2, *p* = 0.854) and interaction effects (F = 1.6, *p* = 0.175). The MT group exhibited maximal improvement magnitude (Δ = 15.22, 95% CI 12.25–18.18). WMFT outcomes followed similar temporal patterns with significant time effects (*p* < 0.001) but no intergroup differences (*p* > 0.05). Both elbow flexion MAS (F = 95.36) and extension MAS (F = 59.17) showed marked time effects (both *p* < 0.001) without significant between-group variations.

Subgroup analysis, as shown in Table 3, identified significant post-intervention elbow extension MAS reduction in Brunnstrom III–IV patients receiving MT and RMT at follow-up. All groups demonstrated muscle tone rebound during follow-up. Brunnstrom staging revealed significant time effects for upper limb (F = 182.08), hand (F = 87.75), and lower limb (F = 63.81) recovery (all *p* < 0.001). The MT group achieved superior hand function recovery versus controls at follow-up (mean difference = 1.04, 95% CI 0.76–1.33). Among Brunnstrom I–II patients, MT showed greater upper limb improvement than controls (*p* = 0.046). BBT scores demonstrated significant time × intervention interaction (F = 0.76, *p* = 0.030), with MT exhibiting maximal gains (Δ = 5.91, 95% CI 3.77–8.06). MBI analysis confirmed notable time effects (F = 123.73, *p* < 0.001), particularly in MT (Δ = 23.43, 95% CI 17.6–29.3).

### 3.3. The Functional Connection of Brain Networks

**Figure 2:**
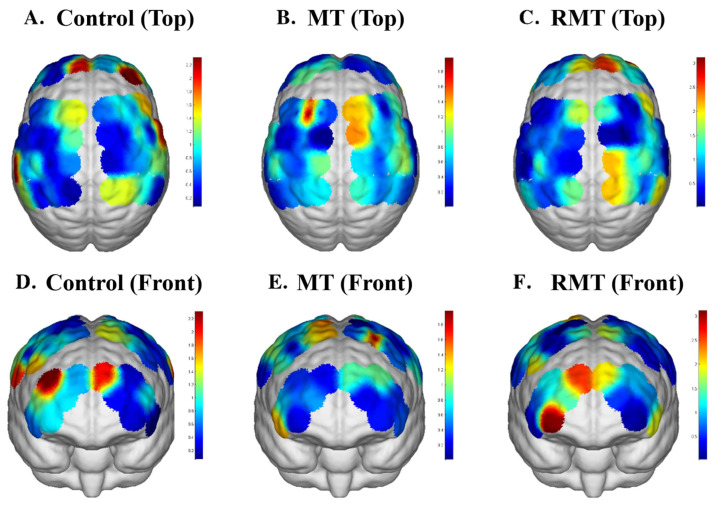
fNIRS activation maps of all channels in the three groups after intervention. Note: Higher HbO_2_ concentration levels are represented by colors closer to red, while lower levels are represented by colors closer to blue.

**Figure 3:**
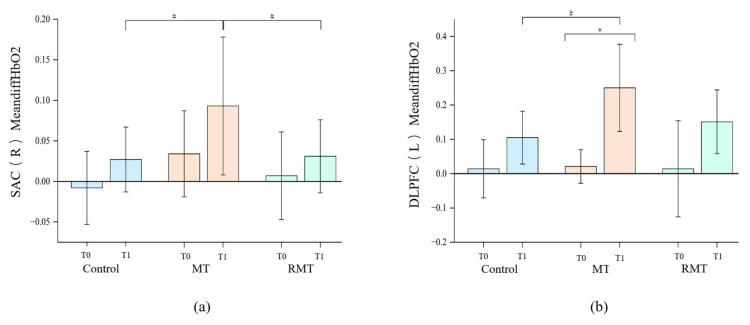
(**a**) SAC, (**b**) DLPFC. The mean difference in HbO_2_ and HbT across brain regions among the three groups. * significant within groups; # significant between groups.

fNIRS data revealed significant between-group differences in oxyhemoglobin concentration changes (Mean_diff_HbO_2_) within the right sensory association cortex (SAC) (*p* = 0.008), with MT outperforming both control (*p* = 0.03) and RMT (*p* = 0.043). Functional connectivity analysis indicated enhanced post-treatment coupling between left dorsolateral prefrontal cortex (DLPFC) and right DLPFC (r = 0.68 vs. 0.41, *p* = 0.012) as well as right SAC (r = 0.59 vs. 0.33, *p* = 0.028) in the MT group. The brain activation topographic maps of the three groups are shown in Figure 2. Table 4 shows the mean differences of HbO_2_ and HbT in each brain region across the three groups. Significant differences in MeandiffHbO_2_ among the three groups were observed in the right SAC (Figure 3a) and left DLPFC (Figure 3b) across all brain regions.

**Figure 4:**
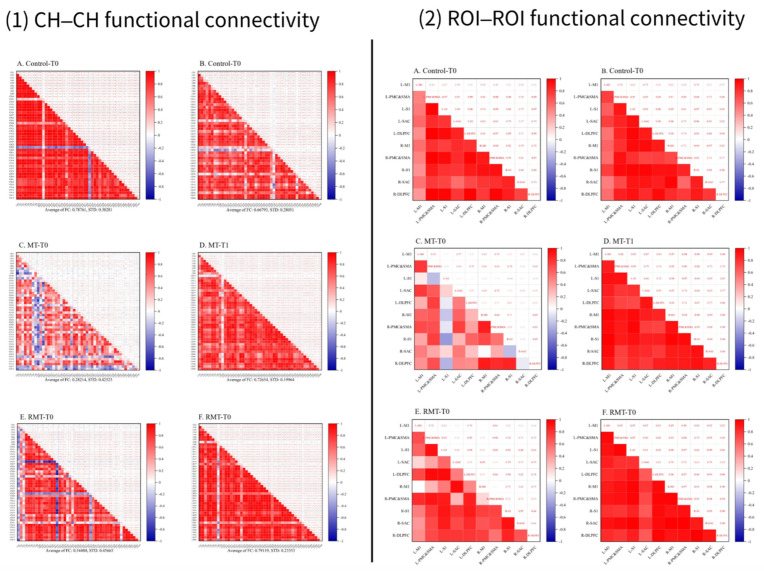
Heatmaps of CH–CH and ROI–ROI functional connectivity for each group. Note: CH–CH refers to channel-to-channel connectivity; the color bar represents the functional connectivity strength (Pearson correlation coefficient) between pairs of channels, with stronger connectivity shown in red and weaker connectivity in blue. ROI–ROI refers to region-to-region connectivity; the color bar represents the functional connectivity strength (Pearson correlation coefficient) between pairs of regions, with stronger connectivity shown in red and weaker connectivity in blue.

In the control group, the mean functional connectivity strength decreased slightly after treatment (0.67 ± 0.28) compared with before treatment (0.79 ± 0.30). In the MT group, it increased significantly from 0.28 ± 0.43 to 0.73 ± 0.20, while in the RMT group, it also increased significantly from 0.54 ± 0.46 to 0.79 ± 0.23. The functional connectivity matrices of CH–CH and ROI–ROI for the three groups are shown in Figure 4.

## 4. Discussion

This study systematically evaluated the clinical efficacy and neural mechanisms of RMT compared with conventional MT and routine rehabilitation in patients with post-stroke upper limb dysfunction. This study systematically evaluated the clinical efficacy and neural mechanisms of RMT compared with conventional MT and routine rehabilitation in patients with post-stroke upper limb dysfunction. Notably, improvements were observed not only in the RMT and MT groups but also in the control group, reflecting the expected spontaneous recovery in subacute stroke as well as the effects of standard rehabilitation. Consequently, it is difficult to attribute all observed changes solely to the type of intervention. Nevertheless, the randomized group × time analyses revealed that the magnitude and pattern of improvements in certain outcomes differed between groups, suggesting that RMT and MT may provide additional benefits beyond spontaneous recovery. Furthermore, fNIRS revealed distinct cortical activation and connectivity patterns associated with MT, providing novel insights into its underlying mechanisms.

This study evaluated the efficacy and mechanisms of robot-assisted mirror therapy (RMT) compared with conventional mirror therapy (MT) and routine rehabilitation in patients with post-stroke upper limb dysfunction. Consistent with the natural history of subacute stroke, motor improvement was observed across all groups, including the control group receiving standard care. This underscores the critical influence of spontaneous neural recovery and baseline rehabilitation. However, our randomized, controlled design and group × time interaction analyses revealed that the magnitude and pattern of improvement diverged significantly between interventions. Specifically, RMT and MT led to greater and/or distinct gains in certain functional and neurophysiological outcomes compared to the control group. These differential trajectories suggest that RMT and MT confer specific therapeutic benefits that augment the course of natural recovery.

### 4.1. Clinical Significance of Functional Outcomes

It is well recognized that the recovery of motor function may be achieved through the combined effect of spontaneous biological processes and “usage-dependent” processes, including motor learning and skill acquisition [38]. To reduce the influence of spontaneous recovery, our randomized design ensured comparable baseline characteristics, identical rehabilitation dosage, and synchronized assessments across all groups. Randomization distributes time-related recovery evenly, so any between-group differences are more likely attributable to the interventions. Our analysis also examined the group × time interaction, which tests whether improvement trajectories differ beyond spontaneous recovery. Although all groups improved, the distinct patterns and magnitudes of change support an additional treatment effect. The observed improvements in FMA-UE, WMFT, MAS, BBT, and Brunnstrom stages indicate that both RMT and MT facilitate recovery of upper limb motor function, particularly fine motor skills and coordination, which are critical for regaining independence. Reduction in muscle tone, as reflected by MAS scores, further suggests that these interventions may mitigate spasticity, thereby supporting smoother voluntary movement. The gains in MBI and SF-36 highlight that beyond motor restoration, MT and RMT also enhance activities of daily living and health-related quality of life. Notably, MT produced more consistent benefits across multiple measures compared with RMT, suggesting that the active engagement and embodiment processes inherent to MT may play a decisive role in functional recovery.

Our study demonstrated significant time effects for FMA-UE, WMFT, Brunnstrom staging, and the BBT in all groups, indicating functional improvement over the intervention period. However, the intervention effect itself was not statistically significant, suggesting that RMT and MT, compared to conventional therapy, may not produce markedly superior improvements in overall upper limb function.

Subgroup analysis revealed that patients in Brunnstrom stages I–II and III–IV both exhibited notable functional gains over time, indicating that early- and mid-stage hemiplegic patients can benefit from conventional rehabilitation, MT, and RMT. Notably, MT produced improvements in FMA-UE and WMFT scores that exceeded the minimal clinically important difference (MCID), particularly enhancing hand dexterity and fine motor control. Conversely, RMT showed moderate improvement in overall upper limb function but less pronounced effects on hand-specific tasks. This discrepancy may relate to the characteristics of the interventions: MT provides visual feedback simulating individualized movement, engaging multiple cortical networks, whereas RMT relies on robotic guidance through predefined movement patterns, limiting flexibility and cognitive engagement.

Consistent with previous studies, RMT has been reported to enhance upper limb function and ADL outcomes post-stroke. For instance, Jin et al. (2024) [39] observed superior improvements in hand function and MBI scores in RMT participants compared to MT and control groups. However, some studies have suggested that the therapeutic effects of RMT did not produce significant differences between groups. Chen et al. (2023) [28] and He et al. (2023) [40] further confirmed the efficacy of RMT in promoting upper limb recovery, although the intergroup differences were not always statistically significant.

Our findings also indicate that all three groups exhibited significant time-dependent reductions in elbow spasticity, suggesting that in-hospital rehabilitation effectively controlled muscle tone. Notably, MT appeared most effective for elbow flexion, while RMT showed greater gains for elbow extension, particularly in participants at Brunnstrom stage III–IV, suggesting that these therapies may offer specific advantages during later stages of upper limb recovery [41].

MT primarily relies on cortical-level modulation: mirror-induced visual illusions activate the mirror neuron system and enhance inhibitory control over the M1, thereby reducing the deficits of flexor muscle synergies in top-down regulation. In contrast, RMT operates predominantly through spinal and peripheral mechanisms. Robotic stretching combined provides continuous low-load traction and rich sensory input to the extensor muscles, reducing spinal reflex excitability. This process particularly beneficial for patients in Brunnstrom stages III–IV who often exhibit pronounced extensor spasticity. Although partial rebound in muscle tone during follow-up suggests that spinal-level modulation alone may be insufficient for long-term cortical reorganization. Overall, MT appears more suitable for patients in earlier recovery phases who retain a certain degree of cortical control, whereas RMT may be advantageous for individuals with more severe cortical impairment who require direct modulation of spinal reflex pathways.

All groups showed significant improvements in Modified Barthel Index (MBI) scores over time, indicating enhanced ADL performance. The MT group exhibited the largest improvement at follow-up compared to pre-intervention (23.43, 95% CI: 17.6–29.3), with RMT and control groups demonstrating smaller gains. These findings suggest that although RMT improves ADL ability, the extent of improvement may be influenced by the content, intensity, and patient engagement in training. Prior studies corroborate these observations; for example, Jan Mehrholz et al. (2015) [42] found that Electromechanical and robot-assisted arm training enhanced finger dexterity and ADL performance. SF-36 scores demonstrated significant time effects in all groups, while intervention and interaction effects were not statistically significant. The MT group showed the smallest decline (−1.74, 95% CI: −3.8–0.32), whereas RMT and control groups experienced slightly larger decreases (−3.36 to −3.39). These results suggest that the interventions did not substantially improve quality of life over the short term, potentially reflecting adjustment and adaptation processes following therapy. Subgroup analysis indicated that patients with higher baseline function (Brunnstrom I–II) adapted more rapidly, maintaining higher quality of life, whereas those with more severe impairments (III–IV) improved more slowly, potentially limiting observable group differences. These findings align with previous reports, indicating that RMT may enhance quality of life, but its effects depend on functional baseline status.

### 4.2. Neural Mechanisms and Implications

Neuroimaging analysis provided converging evidence that MT induced stronger activation in bilateral premotor and parietal regions, along with enhanced functional connectivity between motor-related networks. These regions are critically involved in motor planning, visuomotor integration, and the coordination of complex movements. These findings are consistent with prior studies indicating that MT leverages the mirror neuron system, visuomotor coupling, and attention-motor interactions to promote neuroplasticity. In contrast, RMT primarily elicited activation related to movement execution but showed weaker engagement of higher-order cognitive–motor networks. This suggests that while RMT offers mechanical precision and repetition, it may insufficiently stimulate the cognitive and perceptual processes that drive cortical reorganization. Future optimization of RMT should consider integrating features that foster patient engagement, embodiment, and sensorimotor integration, rather than relying solely on robotic assistance.

fNIRS analysis revealed that MT significantly activated the right SAC, a region critically involved in sensorimotor integration, motor planning, and movement execution [43]. The right SAC integrates sensory inputs with motor commands, enabling coordinated limb movements and fine motor control, and plays a key role in translating cognitive motor plans into precise motor outputs. Enhanced activation in this region likely underpins the observed improvements in FMA-UE and WMFT scores in the MT group, suggesting that MT can promote motor recovery by engaging cortical areas responsible for sensorimotor processing.

The left DLPFC, a region associated with cognitive control, attention, and motor planning [44], showed increased activation in the MT group compared to RMT and control groups. The DLPFC supports higher-order functions such as strategy formulation, adaptive motor planning, and executive control over task performance. This activation may explain the improvements observed in functional outcomes, such as WMFT performance and MBI scores, as enhanced DLPFC activity supports strategy formulation, task execution, and adaptive motor planning [45]. The findings suggest that MT not only engages motor-related regions but also recruits higher-order cognitive control areas, contributing to better motor performance and functional independence.

Functional connectivity analysis demonstrated enhanced connectivity between the left and right DLPFC in the MT group. Strengthened interhemispheric connectivity of the DLPFC may facilitate coordinated cognitive–motor processing, integration of attentional control with motor execution, and efficient execution of complex tasks [44,46], contributing to improvements in activities of daily living. This enhanced network integration highlights MT’s capacity to promote not only localized cortical activation but also distributed cortical network coordination, which is critical for functional recovery post-stroke.

Correlational analysis between clinical measures and fNIRS data further supported the mechanistic link between brain activation and functional recovery. Patients showing greater increases in right SAC activation exhibited more substantial gains in FMA-UE and WMFT scores, while enhanced left DLPFC activation correlated with improvements in ADL performance as measured by MBI. These results underscore the functional significance of the engaged cortical regions: SAC activation facilitates precise sensorimotor control, while DLPFC activation underlies cognitive–motor integration and executive control, collectively driving observable improvements in motor and daily functional outcomes. These findings provide direct evidence that MT-induced neuroplastic changes in specific cortical regions are associated with functional recovery, reinforcing the neurophysiological basis of MT.

Increased activation in the dorsolateral prefrontal cortex is discussed in the context of enhanced cognitive engagement and attentional control necessary for motor learning. Modulation of activity in the premotor and primary motor cortices is interpreted as reflecting adaptations in motor planning and execution circuits. The observed changes in interhemispheric connectivity (e.g., between motor areas) are explicitly tied to theories of reduced cortical inhibition from the non-lesioned hemisphere and improved functional integration, which may underlie the observed motor gains. By framing the neurophysiological findings within these established functional frameworks, we have strengthened the clinical and mechanistic relevance of our fNIRS results.

MT and RMT are fundamentally visual-feedback–driven motor relearning approaches. The repeated practice over three weeks likely induces continuous activation and refinement of internal models, thereby promoting neural reorganization and functional recovery. However, the two interventions appear to differ in the level of embodiment they evoke, which may reflect differences in both the degree and the specific components of the sensorimotor internal model that are engaged [47]. MT may involve greater participation of both inverse and forward internal models, engaging prefrontal–parietal circuits related to action intention as well as the descending motor control pathways spanning the motor cortex-cerebellum-spinal motor neurons [48]. Within the inverse model, the cerebellum plays a critical role by integrating sensory and motor information to accurately predict movement consequences and rapidly adapt when errors occur [49]. There is converging evidence that the cerebellum is essential for implementing internal models [50] and for modulating components of the mirror neuron system [51]. Although the fNIRS used in this study did not include cerebellar regions, the observed changes in motor cortical activation and functional connectivity, together with previous findings regarding cerebellar contributions to inverse model processing [50,52], suggest that MT may more effectively recruit cerebellar circuits during visually guided, cognitively engaged voluntary movements. Such enhanced cerebellar involvement could strengthen sensorimotor integration, facilitate cortical reorganization, and ultimately support more robust cognitive–motor coupling during recovery.

Beyond the hemodynamic measures employed in this study, emerging research highlights several alternative biomarkers that may offer deeper insights into the mirror system’s function. For instance, event-related desynchronization (ERD) of mu/beta rhythms, measured via electroencephalography, provides millisecond-level temporal resolution to track the dynamic suppression of sensorimotor oscillations during action observation and imagery, serving as a sensitive correlate of mirror neuron system engagement [53]. Additionally, corticomuscular coherence or TMS-evoked motor potentials could directly quantify therapy-induced changes in corticospinal excitability and functional connectivity between specific brain regions and spinal motor pools [54]. While the present fNIRS data delineate spatial activation patterns, integrating these complementary modalities in future work—combining fNIRS’s spatial mapping, EEG’s temporal precision, and TMS’s circuit-level probing—could yield a robust, multimodal biomarker framework. Such a framework would not only enhance mechanistic understanding but also potentially identify predictors of treatment response, enabling more personalized rehabilitation strategies for stroke patients.

In summary, MT and RMT may influence the neural pathways of motor recovery with different emphases. MT heavily relies on intact visuomotor integration. By activating the mirror neuron system, it primarily promotes the reorganization of intracortical and interhemispheric inhibitory functions. This may be particularly important for improving abnormal synergy patterns (such as flexor spasticity) and tasks requiring fine cognitive engagement. RMT, while providing mirror visual feedback, adds repetitive, guided limb movement. This robot-assisted active–passive cycle not only provides intensive peripheral proprioceptive input (acting at the spinal level) but may also indirectly influence the cortex through sensorimotor closed loops. Therefore, RMT may have advantages in restoring basic joint mobility, muscle strength, and reducing severe spasticity (especially in extensor muscles), which is crucial for laying the functional foundation in patients at early Brunnstrom stages (III–IV).

Consequently, the finding that RMT did not outperform MT on all measures in this study may reflect the differentiated advantages of the two interventions, rather than a simple difference in efficacy. For patients with relatively preserved cortical inhibitory control, the “top-down” cognitively driven mode of MT might be more efficient; for patients with severely impaired cortical descending drive, the “bottom-up” sensorimotor drive provided by RMT may be more critical. This explanation underscores the importance of personalized treatment selection based on the patient’s specific stage of impairment and pattern of functional deficits. We have clarified this viewpoint in the Discussion.

### 4.3. Strengths and Limitations

The study possesses several strengths: (1) A comprehensive comparison of conventional therapy, MT, and RMT, coupled with fNIRS evaluation, provides a multidimensional understanding of intervention efficacy and neural mechanisms. (2) Multi-dimensional outcome measures—including upper limb function, ADL, and quality of life—combined with cortical activation data, enhance interpretability. (3) Stratified block randomization ensured baseline comparability, reducing confounding. (4) Interventions were clinically feasible, supporting potential translation into practice. Limitations include: (1) A relatively small sample size, potentially reducing statistical power to detect intervention differences. (2) Short intervention duration (three weeks), limiting observation of long-term neuroplasticity and functional recovery. (3) fNIRS can only detect cortical surface activity, precluding assessment of deep structures such as the basal ganglia or brainstem.

Future research should include larger samples, longer intervention periods, and multimodal neuroimaging to better elucidate the efficacy and underlying mechanisms of RMT and MT. In addition, RMT protocols may be improved by greater individualization and by adjusting task complexity to enhance participant engagement and cognitive–motor integration. Studies with expanded samples and standardized acquisition procedures will also be better positioned to apply integrated mixed-effects models that incorporate dimension-reduced neurophysiological predictors.

In conclusion, by integrating clinical and neurophysiological evidence, this study elaborates the distinct benefits of MT and RMT for upper limb recovery post-stroke. MT appears to drive superior functional and cortical network outcomes through visual feedback-mediated neuroplasticity, whereas RMT provides more modest gains, predominantly through robotic-assisted motor guidance. These insights offer a rationale for personalizing rehabilitation strategies to individual patient needs.

## Figures and Tables

**Table 1:** Baseline characteristics of the participants.

Variable	Control (*n* = 22)	MT (*n* = 23)	RMT (*n* = 23)	*p*
Age(year), M [P25, P75]	57.50 [49.50, 60.00]	55.00 [42.00, 58.50]	51.00 [44.00, 60.00]	0.562 ^➀^
Gender, *n* (%)				
Female (reference = Male)	3 (13.6%)	8 (34.8%)	5 (21.7%)	0.24 ^➁^
Marital status, *n* (%)				
Married (reference = Others)	20 (90.9%)	19 (82.6%)	22 (95.7%)	0.338 ^➂^
Degree of education, *n* (%)				0.654 ^➁^
Primary school & below	1 (4.5%)	4 (17.4%)	5 (21.7%)	
Junior high school	9 (40.9%)	5 (21.7%)	5 (21.7%)	
High school	3 (13.6%)	3 (13.0%)	1 (4.3%)	
College	8 (36.4%)	10 (43.5%)	11 (47.8%)	
Master & above	1 (4.5%)	1 (4.3%)	1 (4.3%)	
Stroke types, *n* (%)				
Hemorrhagic (reference = Ischemic)	13 (59.1%)	8 (34.8%)	11 (47.8%)	0.262 ^➂^
lesion location, *n* (%)				0.056 ^➁^
Cortex	7 (31.8%)	4 (17.4%)	7 (30.4%)	
Subcortex	15 (68.2%)	13 (56.5%)	9 (39.1%)	
Mix	0 (0.0%)	6 (26.1%)	7 (30.4%)	
Hemiplegic side, *n* (%)				
Right (reference = Left)	9 (40.9%)	10 (43.5%)	11 (47.8%)	0.894 ^➂^
Disease duration (months), M [P25, P75]	1.20 [0.92, 2.98]	1.30 [0.90, 2.50]	1.00 [0.60, 2.20]	0.6 ^➀^
<3 months, *n* (%)	16 (72.7%)	18 (78.3%)	20 (87.0%)	
≥3 months, *n* (%)	6 (27.3%)	5 (21.7%)	3 (13.0%)	0.491 ^➁^
Brunnstrom stage, *n* (%)				0.812 ^➂^
I–II	14 (63.6%)	13 (56.5%)	15 (65.2%)	
III–IV	8 (36.4%)	10 (43.5%)	8 (34.8%)	
Hypertension, *n* (%)				
Yes (reference = No)	19 (86.4%)	17 (73.9%)	18 (78.3%)	0.579 ^➂^
Hyperlipidemia, *n* (%)				
Yes (reference = No)	6 (27.3%)	5 (21.7%)	6 (26.1%)	0.902 ^➂^
Diabetes, *n* (%)				
Yes (reference = No)	4 (18.2%)	4 (17.4%)	6 (26.1%)	0.724 ^➁^
Smoking, *n* (%)				
Yes (reference = No)	10 (45.5%)	3 (13.0%)	3 (13.0%)	0.013 ^➁^*
Drinking, *n* (%)				
Yes (reference = No)	7 (31.8%)	4 (17.4%)	7 (30.4%)	0.476 ^➁^

Note: MT: Mirror Therapy; RMT: Robot-Assisted Mirror Therapy; M [Px, Px]: Median and interquartile range; *n* (%): Frequency and percentage. Statistical methods: ^➀^ Kruskal–Wallis test, ^➁^ Fisher’s exact test, ^➂^ Chi-square test; * Significant intergroup difference, *p* < 0.05.

**Table 2:** The clinical index scores of the three groups at each time point ($\overset{-}{X}$ ± s).

	Group	T0	T1	T2	Time Effect		Intervention Effect		Interaction Effect	
					F	*p*	F	*p*	F	*p*
FMA-UE	Control	16.23 ± 12.66	23.18 ± 13.03	27.95 ± 15.38	192.2	<0.001 *	0.2	0.854	1.6	0.175
	MT	15.5 ± 11.01	24.74 ± 12.64	30.74 ± 13.89						
	RMT	15.30 ± 12.17	22.35 ± 13.22	27.00 ± 14.34						
WMFT	Control	12.55 ± 12.66	19 ± 11.88	22.45 ± 12.98	185.3	<0.001 *	0.3	0.764	1.65	0.167
	MT	13.96 ± 12.74	21.65 ± 13.78	27.17 ± 14.89						
	RMT	11.78 ± 13.62	20.7 ± 15.34	24.48 ± 16.75						
MAS (elbow fle)	Control	1.77 ± 0.81	1.05 ± 0.9	1.23 ± 0.92	95.36	<0.001 *	0.43	0.65	0.54	0.703
	MT	1.65 ± 0.88	0.78 ± 0.85	0.91 ± 0.9						
	RMT	1.74 ± 0.92	1 ± 1	1.09 ± 0.95						
MAS (elbow ext)	Control	1.77 ± 0.87	1.09 ± 0.87	1.41 ± 0.8	59.17	<0.001 *	2.13	0.127	0.86	0.49
	MT	1.43 ± 0.66	0.7 ± 0.82	1 ± 1						
	RMT	1.39 ± 0.78	0.7 ± 0.93	0.78 ± 1						
Brunnstrom (upper limb)	Control	2.23 ± 1.02	2.82 ± 0.8	3.18 ± 0.73	182.08	<0.001 *	0.74	0.483	2.19	0.074
	MT	2.35 ± 0.78	3.09 ± 0.73	3.65 ± 0.71						
	RMT	2.22 ± 1.09	3.13 ± 1.01	3.52 ± 0.9						
Brunnstrom (hand)	Control	1.86 ± 0.83	2.41 ± 0.8	2.64 ± 0.79	87.75	<0.001 *	0.62	0.543	0.64	0.635
	MT	2 ± 0.9	2.65 ± 0.78	3.04 ± 0.82						
	RMT	2 ± 1.09	2.61 ± 1.03	2.96 ± 1.07						
BBT	Control	1.23 ± 3.21	3.77 ± 5.23	4.59 ± 6.11	33.59	<0.001 *	0.26	0.769	2.76	0.030 *
	MT	1.04 ± 2.53	4.52 ± 5.8	6.96 ± 7.75						
	RMT	2.09 ± 4.7	3.04 ± 6	4.48 ± 7.69						
MBI	Control	57 ± 22.2	67.55 ± 20.35	72.95 ± 17.37	123.73	<0.001 *	0.36	0.7	1.46	0.22
	MT	56 ± 24.06	70.87 ± 19.27	79.43 ± 14.21						
	RMT	51.87 ± 32.09	64.83 ± 28.79	73 ± 23.29						
SF-36	Control	63.5 ± 14	61 ± 14.19	57.64 ± 15.38	22.2	<0.001 *	0.79	0.458	0.83	0.439
	MT	68 ± 18.50	66.35 ± 18	64.61 ± 19.15						
	RMT	66.56 ± 16.50	64.26 ± 16.06	60.87 ± 16.3						

Note: * *p* < 0.05.

**Table 3:** Subgroup Analysis of Clinical Index Score ($\overset{-}{X}$ ± s).

	Group	T0	T1	T2	Time Effect		Intervention Effect		Interaction Effect	
					F	*p*	F	*p*	F	*p*
FMA-UE										
I–II	Control	11 ± 8.69	18 ± 11.1	21.86 ± 13.04	115	<0.001 *	0.59	0.56	1.29	0.28
	MT	10.77 ± 8.04	19.38 ± 10.92	25.92 ± 12.76						
	RMT	8.93 ± 5.27	15.67 ± 6.32	20.4 ± 7.12						
III–IV	Control	25.38 ± 13.79	32.25 ± 11.51	38.62 ± 13.77	70.51	<0.001 *	0.19	0.83	0.54	0.707
	MT	21.7 ± 11.61	31.7 ± 11.68	37 ± 13.32						
	RMT	27.25 ± 12.65	34.88 ± 13.93	39.38 ± 16.64						
WMFT										
I–II	Control	4.71 ± 3.87	11.71 ± 6.53	15 ± 9.32	128.64	<0.001 *	0.64	0.533	0.78	0.542
	MT	6.77 ± 6.14	13.46 ± 6.49	18.85 ± 6.41						
	RMT	4.13 ± 4.14	12.87 ± 7.54	16.2 ± 7.82						
III–IV	Control	26.25 ± 10.74	31.75 ± 7.25	35.5 ± 6.21	56.7	<0.001 *	0.12	0.891	1.07	0.384
	MT	23.3 ± 13.23	32.3 ± 13.61	38 ± 16.02						
	RMT	26.12 ± 13.72	35.38 ± 15.45	40 ± 18.36						
MAS (elbow flexion)										
I–II	Control	1.71 ± 0.83	0.93 ± 0.92	1.14 ± 1.03	61.87	<0.001 *	0.12	0.887	0.65	0.626
	MT	1.69 ± 0.85	0.77 ± 0.83	0.85 ± 0.9						
	RMT	1.67 ± 0.9	0.93 ± 1.03	1.07 ± 0.96						
III–IV	Control	1.88 ± 0.83	1.25 ± 0.89	1.38 ± 0.74	31.28	<0.001 *	0.41	0.668	0.38	0.822
	MT	1.6 ± 0.97	0.8 ± 0.92	1 ± 0.94						
	RMT	1.88 ± 0.99	1.12 ± 0.99	1.12 ± 0.99						
MAS (elbow extension)										
I–II	Control	1.64 ± 0.84	0.93 ± 0.83	1.36 ± 0.84	25.75	<0.001 *	0.21	0.814	0.61	0.654
	MT	1.46 ± 0.66	0.92 ± 0.86	1.15 ± 1.07						
	RMT	1.47 ± 0.83	0.87 ± 0.99	1 ± 1.07						
III–IV	Control	2 ± 0.93	1.38 ± 0.92	1.5 ± 0.76	39.6	<0.001 *	3.79	0.038 *	1.33	0.271
	MT	1.4 ± 0.7	0.4 ± 0.7	0.8 ± 0.92						
	RMT	1.25 ± 0.71	0.38 ± 0.74	0.38 ± 0.74						
Brunnstrom (upper limb)										
I–II	Control	1.57 ± 0.51	2.36 ± 0.5	2.79 ± 0.58	181.04	<0.001 *	1.82	0.176	1.21	0.312
	MT	1.77 ± 0.44	2.62 ± 0.51	3.31 ± 0.48						
	RMT	1.53 ± 0.52	2.53 ± 0.64	3 ± 0.53						
	Control	3.38 ± 0.52	3.62 ± 0.52	3.88 ± 0.35						
III–IV	MT	3.1 ± 0.32	3.7 ± 0.48	4.1 ± 0.74	37.63	<0.001 *	3.17	0.061	1.77	0.151
	RMT	3.5 ± 0.53	4.25 ± 0.46	4.5 ± 0.53						
Brunnstrom (hand)										
	Control	1.43 ± 0.51	2.07 ± 0.73	2.43 ± 0.85						
I–II	MT	1.54 ± 0.52	2.31 ± 0.48	2.77 ± 0.73	73.48	<0.001 *	0.93	0.4	0.29	0.883
	RMT	1.33 ± 0.49	2.07 ± 0.8	2.4 ± 0.63						
	Control	2.62 ± 0.74	3 ± 0.53	3 ± 0.53						
III–IV	MT	2.6 ± 0.97	3.1 ± 0.88	3.4 ± 0.84	19.59	<0.001 *	0.1	2.535	0.99	0.425
	RMT	3.25 ± 0.71	3.62 ± 0.52	4 ± 0.93						
BBT										
	Control	0 ± 0	2.07 ± 3.6	3.14 ± 5.5						
I–II	MT	0.69 ± 1.97	2.46 ± 4.72	4.08 ± 6.58	10.71	<0.001 *	2.08	0.14	1.6	0.183
	RMT	0 ± 0	0 ± 0	0.67 ± 1.8						
	Control	3.38 ± 4.75	6.75 ± 6.48	7.12 ± 6.64						
III–IV	MT	1.5 ± 3.17	7.2 ± 6.2	10.7 ± 7.85	29	<0.001 *	0.53	0.6	2.2	0.08
	RMT	6 ± 6.52	8.75 ± 7.48	11.62 ± 9.49						
MBI										
	Control	49 ± 21.16	60 ± 19.12	66.93 ± 17.12						
I–II	MT	46.08 ± 18.23	61.54 ± 15.4	72.92 ± 12.57	105.38	<0.001 *	0.76	0.477	1.53	0.2
	RMT	37.67 ± 27.93	52.93 ± 27.6	63.87 ± 23.07						
	Control	71 ± 17.16	80.75 ± 15.81	83.5 ± 12.71						
III–IV	MT	68.9 ± 25.39	83 ± 17.39	87.9 ± 11.9	29.84	<0.001 *	0.4	0.673	0.82	0.516
	RMT	78.5 ± 20.82	87.12 ± 14.52	90.12 ± 11.26						
SF-36										
	Control	60 ± 12.50	57.71 ± 12.39	53.43 ± 12.74						
I–II	MT	60.75 ± 17.22	63.77 ± 16.88	61 ± 18.33	30.93	<0.001 *	1.27	0.293	0.47	0.632
	RMT	59.2 ± 11.80	56.6 ± 11.62	52.67 ± 11.37						
	Control	60.88 ± 16.50	66.75 ± 16.11	65 ± 17.64						
III–IV	MT	71.36 ± 20.57	69.7 ± 19.75	69.3 ± 20.14	1.66	0.21	0.99	0.388	0.26	0.771
	RMT	80.07 ± 13.51	78.62 ± 13.38	76.25 ± 12.74						

Note: * *p* < 0.05.

**Table 4:** MeandiffHbO_2_ and MeandiffHbT in each brain region across the three groups.

	Group	T0	T1	*P*_time_	*P*_intervention_	*P*_interaction_
R-M1						
MeandiffHbO_2_	Control	0.057 ± 0.077	0.011 ± 0.039			
	MT	0.005 ± 0.053	0.036 ± 0.064	0.741	0.173	0.604
	RMT	−0.003 ± 0.12	0.027 ± 0.047			
MeandiffHbT	Control	0.092 ± 0.186	−0.014 ± 0.056			
	MT	−0.018 ± 0.054	0.008 ± 0.121	0.686	0.168	0.373
	RMT	−0.023 ± 0.152	0.003 ± 0.088			
L-M1						
MeandiffHbO_2_	Control	0.012 ± 0.096	0.029 ± 0.022			
	MT	−0.018 ± 0.085	0.045 ± 0.042	0.858	0.271	0.595
	RMT	0.019 ± 0.089	0.008 ± 0.051			
MeandiffHbT	Control	0.053 ± 0.091	0.019 ± 0.041			
	MT	−0.037 ± 0.109	0.047 ± 0.087	0.962	0.066	0.495
	RMT	0.004 ± 0.099	0.008 ± 0.057			
R-PMC&SMA						
MeandiffHbO_2_	Control	0.039 ± 0.088	0.066 ± 0.099			
	MT	0.041 ± 0.156	0.055 ± 0.109	0.953	0.911	0.901
	RMT	0.063 ± 0.188	0.075 ± 0.096			
MeandiffHbT	Control	0.032 ± 0.189	0.021 ± 0.102			
	MT	−0.004 ± 0.156	0.045 ± 0.153	0.064	0.987	0.803
	RMT	0.041 ± 0.213	0.069 ± 0.083			
L-PMC&SMA						
MeandiffHbO_2_	Control	0.047 ± 0.121	0.011 ± 0.058			
	MT	0.085 ± 0.183	0.082 ± 0.097	0.519	0.739	0.274
	RMT	0.056 ± 0.148	0.012 ± 0.107			
MeandiffHbT	Control	0.041 ± 0.168	0.03 ± 0.096			
	MT	0.058 ± 0.224	0.112 ± 0.127	0.681	0.589	0.392
	RMT	0.061 ± 0.212	−0.018 ± 0.168			
R-S1						
MeandiffHbO_2_	Control	0.014 ± 0.085	0.031 ± 0.059			
	MT	0.025 ± 0.093	0.071 ± 0.067	0.885	0.764	0.401
	RMT	0.02 ± 0.09	0.029 ± 0.088			
MeandiffHbT	Control	0.002 ± 0.135	0.022 ± 0.079			
	MT	−0.006 ± 0.101	0.082 ± 0.109	0.145	0.700	0.433
	RMT	0.014 ± 0.116	0.039 ± 0.083			
L-S1						
MeandiffHbO_2_	Control	−0.009 ± 0.124	0.016 ± 0.057			
	MT	0.047 ± 0.105	0.068 ± 0.081	0.989	0.971	0.245
	RMT	0.048 ± 0.09	0.026 ± 0.073			
MeandiffHbT	Control	0.02 ± 0.073	0.002 ± 0.092			
	MT	0.033 ± 0.138	0.079 ± 0.103	0.979	0.701	0.434
	RMT	0.053 ± 0.104	0.016 ± 0.078			
R-SAC						
MeandiffHbO_2_	Control	−0.008 ± 0.045	0.027 ± 0.040			
	MT	0.034 ± 0.053	0.093 ± 0.085	0.529	0.008 *	0.610
	RMT	0.007 ± 0.054	0.031 ± 0.045			
MeandiffHbT	Control	−0.023 ± 0.089	0.05 ± 0.084			
	MT	−0.019 ± 0.073	0.041 ± 0.072	0.582	0.491	0.100
	RMT	0.008 ± 0.095	0.018 ± 0.046			
L-SAC						
MeandiffHbO_2_	Control	0.021 ± 0.061	0.015 ± 0.045			
	MT	0.001 ± 0.057	0.051 ± 0.049	0.840	0.214	0.494
	RMT	−0.001 ± 0.08	0.01 ± 0.064			
MeandiffHbT	Control	0.005 ± 0.05	0.018 ± 0.051			
	MT	−0.017 ± 0.075	0.055 ± 0.057	0.823	0.102	0.210
	RMT	−0.012 ± 0.09	−0.022 ± 0.093			
R-DLPFC						
MeandiffHbO_2_	Control	0.033 ± 0.132	0.057 ± 0.087			
	MT	0.109 ± 0.172	0.046 ± 0.267	0.653	0.709	0.778
	RMT	0.057 ± 0.125	0.054 ± 0.104			
MeandiffHbT	Control	0.052 ± 0.171	0.028 ± 0.082			
	MT	0.074 ± 0.13	0.009 ± 0.37	0.569	0.628	0.854
	RMT	0.042 ± 0.158	0.098 ± 0.155			
L-DLPFC						
MeandiffHbO_2_	Control	0.014 ± 0.085	0.105 ± 0.077			
	MT	0.021 ± 0.049	0.250 ± 0.127	0.042 *	0.059	0.101
	RMT	0.014 ± 0.140	0.151 ± 0.093			
MeandiffHbT	Control	0.022 ± 0.09	0.012 ± 0.07			
	MT	0.141 ± 0.221	0.106 ± 0.104	0.579	0.474	0.076
	RMT	0.1 ± 0.123	0.064 ± 0.09			

Note: * *p* < 0.05.

## Data Availability

The data presented in this study are not publicly available due to restrictions imposed by the ethical approval and the informed consent process, which explicitly state that the data will only be used for the purposes of this research. The data are available on reasonable request from the corresponding author, subject to approval from the institutional ethics committee.
